# The voluntary utilization of visual working memory

**DOI:** 10.1038/s41598-024-58685-5

**Published:** 2024-04-05

**Authors:** Shalva Kvitelashvili, Yoav Kessler

**Affiliations:** Department of Psychology and School of Brain Sciences and Cognition, Ben-Gurion University of the Negev, Beer Sheva, Israel

**Keywords:** Psychology, Human behaviour

## Abstract

While a vast amount of research has focused on understanding the capacity limits of visual working memory (VWM), little is known about how VWM resources are employed in unforced behavior and how they correlate with individual capacity constraints. We present a novel, openly available, and easy-to-administer paradigm enabling participants to freely utilize their VWM capacity. Participants had to reconstruct an array of colored squares. In each trial, they were allowed to alternate between the memory array and the reconstruction screen as many times as they wished, each time choosing how many items to reconstruct. This approach allowed us to estimate the number of utilized items, as well as the accuracy of the reconstruction. In addition, VWM capacity was measured using a change detection task. In two experiments, we show that participants tend to under-utilize their VWM resources, performing well below their capacity limits. Surprisingly, while the extent to which participants utilized their VWM was highly reliable, it was uncorrelated with VWM capacity, suggesting that VWM utilization is limited due to strategic considerations rather than capacity limits.

## Introduction

Visual working memory (VWM) capacity corresponds to the individual's cognitive ability to temporarily retain visual information for short durations. VWM capacity varies substantially among individuals^1^ and is highly correlated with a broad range of cognitive abilities^2,3^. Consequently, it has become a central topic in cognitive psychology research, with an emphasis on benchmarking and characterizing the factors that restrict retention. These studies typically employ controlled paradigms, where participants are required to retain memory items over a few seconds and are then tested on their memoranda^4–8^. This general paradigm is geared toward examining VWM capacity limits during maximal performance, in which the participants are required to maintain as much information as possible. However, this approach overlooks the natural utilization of VWM capacity in spontaneous behavior, in which maximal capacity utilization is not necessarily required and may even be sub-optimal. The present study aims to understand how individuals harness their VWM capacity during unforced behavior, in which they are free to choose the amount of retained information. Furthermore, we examine whether memory utilization is associated with individual differences in VWM capacity.

An early attempt to characterize the utilization of VWM capacity in unforced behavior originated from a series of experiments conducted by Ballard et al.^9^, designed to assess VWM capacity usage within the context of hand–eye coordination tasks. Across a set of computerized and physical experiments, participants were tasked with reproducing a target-model composed of various colored blocks from a pool of available component parts. Crucially, the target-model remained continuously visible, allowing participants to gaze upon it as they wished. This granted participants control over the amount of information they retained in their working memory as they attempted to reproduce the model. By tracking both the eye movements and hand motions of participants during the reproduction process, the researchers revealed a substantial under-utilization of VWM capacity. Specifically, even during the placement of a single block, participants frequently shifted their gaze back to the target-model after picking up the block from the pool of components prior to situating it in its intended location. This behavior indicated a sequential encoding of individual features. Furthermore, the finding suggested that the under-utilization of VWM capacity was a deliberate choice. When the demand for viewing the target-model was heightened, participants reduced the frequency of gaze shifts and leaned more heavily on their VWM resources.

Subsequent to this work, a number of follow-up studies have embraced the approach of gauging VWM utilization through the monitoring of eye and/or hand movements in tasks that allow individuals to control the load on their VWM capacity^10–12^. For instance, Draschkow et al.^10^ implemented a comparable model-copying task, albeit in a virtual reality setting, that facilitated precise gaze tracking. Their results supported the notion that individuals prefer to repeatedly sample their surroundings, rather than fully maximizing their available VWM capacity. This preference persisted even when it led to a noticeable increase in the time required to complete the task. The researchers also replicated the finding that this tendency for frequent sampling can be curbed by increasing the physical effort necessary for environmental sampling. This suggests that participants aim to balance between the cognitive effort needed to load information into VWM and the physical effort associated with sampling the environment. Droll and Hayhoe^12^ further demonstrated that participants optimized their VWM utilization to the complexity of the memoranda. In a brick sorting task where participants had control over their VWM resource utilization, they observed a notable decrease in the number of maintained items, measured by eye tracking, as the bricks were defined by more distinct features. Specifically, participants favored frequent environmental sampling when the bricks consisted of four features, while this inclination diminished when the block contained only two features.

The main aim of investigating how VWM capacity is used in unforced behavior is to understand how people naturally use their memory for visual information in real-life situations. While this research is valuable for insights into everyday reliance on VWM capacity, the methods used are very different from those used in typical research on VWM limitations. First, the stimuli that were used in the aforementioned studies are quite different from those employed in “standard” VWM capacity tasks, often being 2D colored squares (see^6^). Second, the above studies incorporate a visual search component, which may also draw VWM capacity resources^13^. Specifically, reconstructing a model with physical bricks requires the participant to search within the pool of “candidate” bricks before finding and placing the desired one. The involvement of VWM in this search might have limited VWM utilization in these tasks, potentially leading to an underestimation of VWM utilization. Lastly, the methods used are often complex and involve special equipment for tracking both hand and eye movements. This complexity makes these studies less accessible, more expensive, and time-consuming to carry out.

The present study aims to achieve three primary objectives. Firstly, we seek to develop a paradigm that effectively addresses the aforementioned limitations of prior research methodologies. Our computerized paradigm uses 2D colored squares as in many standard VWM experiments, and allows participants to freely utilize their VWM capacity in an unconstrained manner while also providing the researcher with tools to estimate utilization and accuracy metrics, without the need for a virtual reality apparatus or an eye-tracking device. This makes our paradigm suitable for online testing and large-scale individual differences studies. Secondly, the present study aims to investigate whether individuals consistently opt to underutilize their VWM capacity resources, as observed in previous research. Lastly, this research aims to explore the potential correlation between the extent of VWM capacity utilization and an individual's capacity limits.

**Figure 1:**
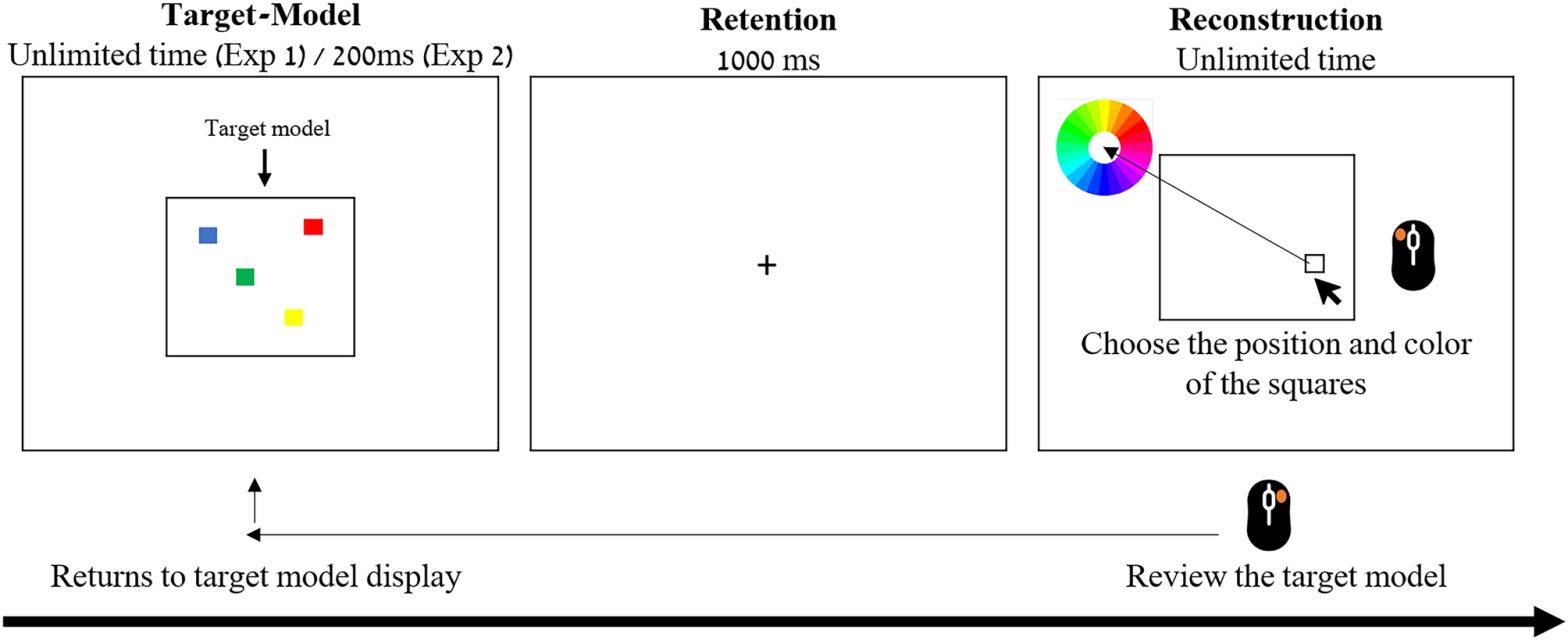
Model Reconstruction Task. Each trial started with a display of the target-model for an unlimited time (Exp1) or for 200 ms (Exp2). The target-model was comprised of either 1, 2, or 4 randomly generated colored squares (i.e., set-size). Following a left click of the computer mouse (or after 200 ms in Exp2) a retention interval appeared for 1000 ms. Finally, the participant entered the reconstruction phase of the task where he was asked to recreate the target-model the best of his abilities by first pressing inside the empty frame to indicate the location of the square, and then selecting the desired color on the color wheel that popped u*p* immediately after the location selection. Participants could press the right mouse-button to review the target-model for an unlimited number of times. Participants were unable to change items that were already placed. The trial finished automatically after the participants placed all the items.

In pursuit of these objectives, we introduce a novel paradigm termed the 'model-reconstruction' task (see Fig. 1). In this task, participants are tasked with recreating a 'target-model' comprised of a randomized arrangement of colored squares. Initially, the model is presented to participants, after which they proceed to the reconstruction phase. During this phase, they are provided with an empty black frame. To recreate the model, participants used the computer mouse to indicate both the position and the color of each of the squares. Critically, participants have the option to freely review the model by pressing a button and to alternate between the model and the reconstruction screen as they wish. By tracking the number of item positions after each review of the model, we can estimate the utilization of VWM capacity in each step. Importantly, this task has been intentionally structured to resemble the stimuli and structure of the delayed estimation paradigm, widely employed to reliably assess VWM capacity limits^14^. In addition to our new tasks, the participants were tested with a visual change detection task^15^ to enable us to examine the correlation between VWM capacity, as measured in standard tasks, and VWM utilization and accuracy in our model reconstruction task.

## Experiment 1

### Method

#### Participants and procedure

Thirty participants (25 females, *M*_*age*_ = 23.3, *SD*_*age*_ = 1.12) were recruited from the student pool at Ben-Gurion University of the Negev. Participants volunteered for the study in exchange for course credit. All participants completed both tasks within a single 1-h session. The study was approved by the Ben-Gurion University Psychology Department's ethics committee in accordance with the Declaration of Helsinki. Informed consent was obtained from all participants. The full data, along with the pre-processing and statistical analysis scripts, are freely available in our paper’s OSF repository (https://osf.io/3cb2k/).

#### Model reconstruction task

The task was programmed using OpenSesame^16^ and is openly available along with a simple pre-processing and analysis script in the paper’s OSF repository (https://osf.io/3cb2k/). Participants commenced the study by completing the model reconstruction task, which lasted approximately 40 min. Within this task, participants were required to manually reconstruct a target-model composed of either 1, 2, or 4 (set-size, SS) colored squares in a free manner. Participants underwent 6 training sequences (two for each SS) followed by 90 test sequences (30 for each SS). Each sequence began with the display of the target-model for an unrestricted duration. The target-model comprised colored squares (0.85° × 0.85°, assuming a 60 cm viewing distance) enclosed within a black frame (13.47° × 13.47°). The colors of the squares were randomly selected from a list of predefined 256 colors sampled from the HSV color space with varying levels of evenly spaced-out Hue angles and a maximal (100) value of Saturation and Value. Square locations were randomly generated, ensuring no overlaps. Participants could view the target-model for as long as they wanted and toggled between the memory and reconstruction phase by pressing the left mouse button key. The reconstruction screen was displayed following a 1000 ms retention interval after the key press. In this screen, they recreated the target-model by selecting the position and color of each colored square. Each time, they chose a square’s location by left-clicking the computer mouse within the frame. After positioning, a continuous color wheel appeared, prompting the selection of a corresponding color for the square. The X–Y coordinates of where the participant clicked on the color wheel were converted to a hue angle. Importantly, participants were afforded the option to review the target-model an unlimited number of times by pressing the right mouse button. There were no penalties or limitations associated with this review process. In each step, the squares that were already placed before appeared on the reconstruction screen, but their position or color could not be changed.

##### VWM capacity utilization estimation

To estimate the utilization of VWM capacity, we assumed that if an item was successfully encoded into VWM, the participant would position it in the reconstruction screen without reviewing the model. Consequently, VWM capacity utilization was the mean number of items placed after each view of the model. Accordingly, the average VWM utilization was calculated by dividing the trials' SS by the number of instances the participant viewed the model.

##### Accuracy measurement

To estimate the accuracy of item placement and color selection, we need to identify which item in the model corresponded to each of the items that were placed in the reconstruction phase. For example, imagine a situation where the model included two items, and the participant placed one item in the reconstruction phase. To measure the accuracy of both the location and the color of the reconstructed item, we needed to know which of the items in the model the participant intended to reconstruct. To do so, we measured the Euclidean distance from the center of each placed item to the centers of all original items in the target-model. The placed item closest in distance to an original item was deemed the participant's attempt at recreation. In instances where different placed items corresponded to the same target-model item, accurate assessment was unfeasible. Consequently, we excluded these trials from the analysis. After establishing correspondence between placed and target-model items, we estimated the accuracy by calculating the amount of error both in the positioning and the color selection. Position error was evaluated by computing the Euclidean distance between the centers of the placed item and its corresponding item in the target-model. Color accuracy was measured as the absolute difference in in radians between the placed item and its corresponding item in the target-model.

#### Change detection task

For the estimation of individual VWM capacity, we employed the change detection task, as utilized in previous studies (e.g.^1,17^). Participants underwent approximately 15 practice trials, followed by 150 test trials. In each trial of this task, a memory array of either SS = 4 or SS = 8 was briefly displayed for 200 ms. The memory array consisted of colored squares (1.37° × 1.37°) encompassing eight highly distinguishable colors: black, blue, brown, cyan, green, magenta, orange, red, and yellow (RGB values: 0,0,0; 68,114,196; 128,64,0; 0,255,255; 0,176,80; 255,0,254; 255,128,65; 254,0,0; 255,255,0). After a retention interval of 1000 ms, a probe screen appeared featuring a single square. Participants were required to indicate whether the color of the square was the same or different compared to the square that occupied the same position in the memory array using the “k” and “s” keys of the keyboard. The key mapping was counterbalanced between participants. In half of the trials, the color was different, while in the remaining half, it remained the same.

VWM capacity was assessed individually for each SS condition using Cowan’s K [4]: K = N*(H − FA). In this equation, K signifies VWM capacity, N stands for the SS, H denotes the hit rate (correct response in change trials), and FA represents the false-alarm rate (incorrect response in no-change trials). The final capacity estimation for each participant was derived as the mean K value across the two SS conditions.

### Results

#### Performance in the change detection task

**Figure 2:**
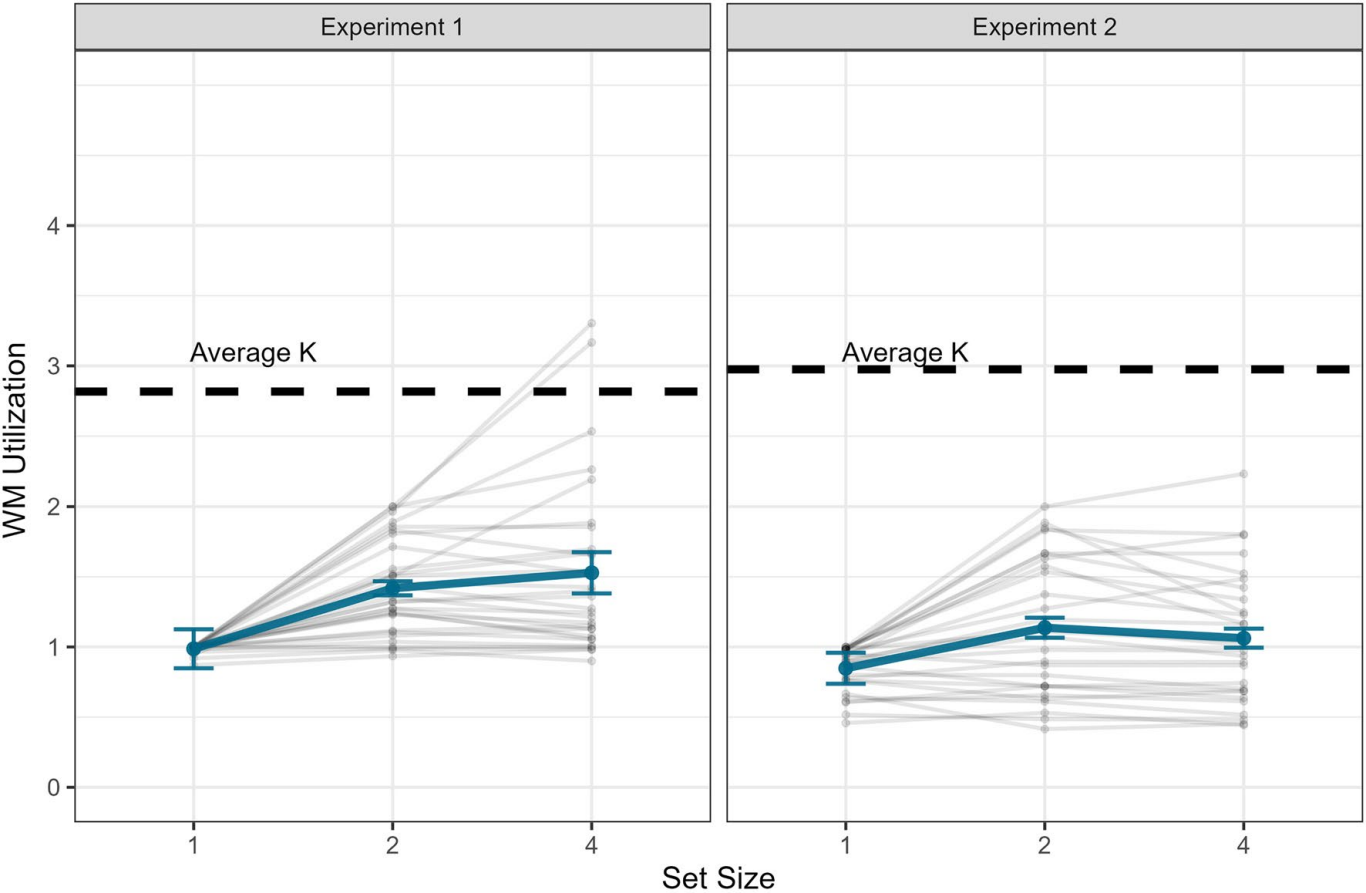
VWM capacity mean utilization in the model-reconstruction task across different set-sizes. Error bars represent 95% within-subject CIs. The dashed line in each panel represents the Average VWM capacity in the change detection task in that experiment.

Trials in which participants’ reaction times (RTs) exceeded ± 3 standard deviations were removed (N trials removed: M = 2.93, SD = 1.41). The mean K was 2.82 (95% CI [2.49, 3.14], SD = 0.903). The Spearman-Brown corrected split-half reliability, measured by calculating the K for odd and even trials separately, was 0.78 (Fig. 2).

#### Performance in the model reconstruction task

Trial sequences in which we were unable to create a direct correspondence between placed and existing items in the target-model were removed from the analysis (1.56% of trials were removed on average SS2, and 9.78% for SS4). Afterwards, trial sequences in which participants had position and color errors that exceeded three standard deviations were also excluded from the analysis (3.44% trials were removed on average for SS1, 7.21% trials for SS2, and 13.3% trials for SS4). Post these exclusions, the analysis comprised 29 (SD = 0.765), 27.4 (SD = 1.1), and 23.5 (SD = 2.18) sequences on average per participant for SSs 1, 2, and 4, respectively.

##### VWM utilization

Mean VWM utilization estimates were 0.987 (within-subject 95% CI [0.848, 1.126]), 1.418 [1.368, 1.468], and 1.529 [1.381, 1.676] for SSs 1, 2, and 4, respectively (F(2, 58) = 23.63, *p* < 0.001, η^2^ = 0.45). Post-hoc contrasts showed that the difference between SS1 and both SS2 and SS4 was significant (t(29) = 5.843, *p* < 0.001, η^2^ = 0.54), while the differences between SS2 and SS4 was non-significant (t(29) = 1.625, *p* = 0.115, η^2^ = 0.08). See Fig. 2. Note that the mean utilization estimate in SS1 was lower than 1 due to the fact that in some trials, participants switched from the target array back to the model without placing the memory item for another view of the model. We also analyzed mean VWM utilization after excluding steps in which participants did not place any items in the reconstruction screen. This analysis aimed to examine whether the low utilization estimates were due to steps where no items were positioned. The estimates of VWM utilization were 1 (within-subject 95% CI [0.86, 1.14]), 1.433 [1.38, 1.48], and 1.56 [1.41, 1.71] for SSs 1, 2, and 4, respectively (F(2, 58) = 24.24, *p* < 0.001, η^2^ = 0.46), very similar to those obtained in the main analysis.

##### Task reliability

We calculated the Spearman-Brown corrected split-half reliabilities, based on odd vs. even trials, for all the dependent measures—utilization, color accuracy, and position accuracy (see Table 1). In addition, since one of our goals was to develop the paradigm as a tool for individual differences research, we wanted to examine the effect of shortening the task in half on reliability. Accordingly, the reliability in the first 15 trials of each SS is reported as well. As can be seen in Table 1, individual differences in VWM utilizations are highly reliable, even with only 15 trials per SS. Since larger set-sizes give rise to more variance in utilization, our results show that VWM utilization can be measured with high reliability based on the SS4 condition alone with merely 15 trials.

**Table 1:** Model reconstruction task reliability. Spearman-Brown corrected split-half reliabilities, based on odd vs. even trials, for all the dependent measures—utilization, color accuracy and position accuracy.

Experiment	All trials			First 15 trials per SS		
	SS1	SS2	SS4	SS1	SS2	SS4
1						
Utilization	0.85	0.97	0.97	0.756	0.943	0.945
Color accuracy	0.392	0.707	0.538	0.134	0.513	0.633
Position accuracy	0.873	0.855	0.947	0.725	0.761	0.729
2						
Utilization	0.964	0.979	0.972	0.936	0.96	0.916
Color accuracy	0.646	0.763	0.858	0.06	0.465	0.809
Position accuracy	0.888	0.894	0.972	0.816	0.643	0.902

##### Model viewing times

Model viewing times were 3365 ms [3040, 3690], 4029 ms [3916, 4141], and 4629 ms [4243, 5016] for SS1, SS2 and SS4, respectively, F(2, 58) = 17.68, *p* < 0.001, η^2^ = 0.38. Post-hoc pairwise contrasts showed that the difference between SS1 and both SS2 and SS4 was significant (t(29) = 4.908, *p* < 0.001, η^2^ = 0.45), and the differences between SS2 and SS4 were also significant (t(29) = 2.866, *p* = 0.008, η^2^ = 0.22).

##### Item positioning and color selection accuracy

Separate repeated-measures ANOVAs indicated that SS had no significant effect on the accuracy of color report (F(2, 58) = 1.88, *p* = 0.161, η^2^ = 0.06) or item positioning (F(2, 58) = 2.34, *p* = 0.105, η^2^ = 0.07).

#### Individual differences

**Figure 3:**
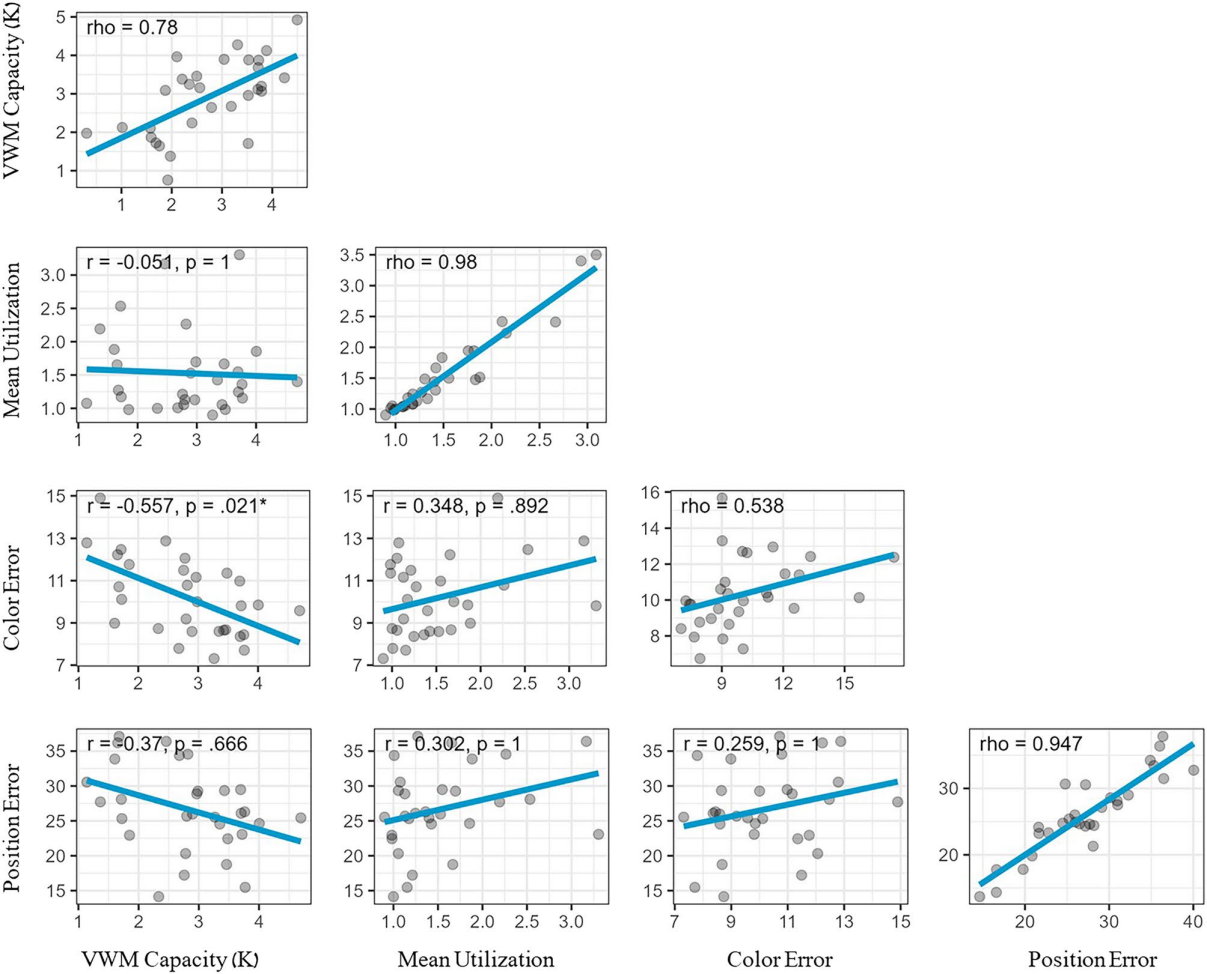
Correlation Matrix from Experiment 1 (SS4 trials only). The VWM capacity was assessed using the change detection task, while VWM utilization, item positioning error, and item color selection error were measured in the model reconstruction task. Each data point represents an individual participant. The main diagonal indicates the Spearman-Brown split-half reliability score (rho) for each metric based on odd vs. even trials.

To explore the relationship between individual VWM capacity and performance in the model reconstruction, Pearson correlations were computed between VWM capacity, VWM utilization, and position and color selection accuracy (Fig. 3). The analysis focused on trials from SS4, which exhibited the greatest between-subject variation in utilization. P-values were Bonferroni-adjusted to the number of correlations. Individual VWM capacity was uncorrelated with utilization (r = − 0.051, p = 1) and with item positioning (r = − 0.37, p = 0.666), but was negatively correlated with color selection error, indicating that participants with higher VWM capacity were more accurate (r = − 0.557, p = 0.021). Lastly, despite being highly reliable (split-half correlation = 0.98), VWM utilization did not significantly correlate with any of the accuracy metrics.

### Discussion

The model reconstruction task proved to be a reliable platform for estimating VWM utilization, as reflected by very high split-half reliability estimates. Furthermore, our findings indicate that, on average, participants tended to under-utilize their VWM capacity while completing the model reconstruction task compared to their performance in the change detection task. In terms of VWM utilization analysis, we observed that participants displayed consistency in the amount of VWM resources they utilized across the task, yet substantial individual differences in utilization levels were evident. While we initially hypothesized that these variations in VWM utilization might stem from differences in VWM capacity limits, we were surprised to find that VWM utilization in the model reconstruction task exhibited no correlation with individual VWM capacity limits. Nevertheless, we unveiled a significant association between VWM capacity limits and color selection accuracy, indicating that higher capacity limits correlated with greater accuracy. This finding suggests that although VWM capacity limits may not directly reflect an individual's inclination to utilize these resources for holding more items in mind, they may indeed indicate an individual's ability to recall these items.

## Experiment 2

The goals of experiment 2 were two-fold. First, we wanted to replicate the findings of Experiment 1, particularly the tendency of participants to under-utilize their VWM capacity and the lack of correlation between VWM capacity and mean utilization. Secondly, we aimed to bring the task closer to the standard change detection task, in which the memory array is typically presented briefly, unlike Experiment 1, in which the target-model was presented for an unlimited time. Accordingly, the presentation time in this experiment was shortened to 200ms.

### Method

#### Participants and procedure

Twenty-eight participants (24 female, *M*_*age*_ = 22.9, *SD*_*age*_ = 0.9) were recruited from the student pool at Ben-Gurion University of the Negev to take part in the study in exchange for course credit. The initial participant count was thirty; however, one participant was excluded due to a negative VWM capacity estimation in the change detection task, and another participant was removed for a high number of errors in the model reconstruction task (Z = 4.66). All participants completed both tasks within a single 1-h session. The session commenced with participants signing an informed consent form and providing demographic information. Subsequently, participants performed the model reconstruction task, followed by the change detection task. The study was approved by the Ben-Gurion University Psychology Department's ethics committee in accordance with the Declaration of Helsinki. Informed consent was obtained from all participants.

The experimental procedure mirrored that of Experiment 1, with the key distinction being the duration of the target-model display in the model reconstruction task. Specifically, in Experiment 2, the target-model was presented for 200 ms instead of an unlimited time, as in Experiment 1 (refer to Fig. 1). Notably, participants retained the option to review the target-model without any restrictions, just as in the previous experiment.

### Results

#### Performance in the change detection task

Trials in which participants’ reaction times (RTs) exceeded ± 3 standard deviations were removed (N trials removed: M = 2.9, SD = 1.06). The mean K was 2.98 (95% CI [2.71, 3.24], N = 28, SD = 0.711), with a Spearman-Brown split-half reliability score of 0.614 (Fig. 2). The VWM capacity estimation did not significantly differ from the estimation of the previous experiment (t(56) = − 0.737, *p* = 0.464, d = − 0.2).

#### Performance in the model reconstruction task

Trial sequences in which we were unable to create a direct correspondence between placed and existing items in the target-model were removed from the analysis completely (3.33% of trials were removed on average SS2, and 10.8% for SS4). Afterward, trial sequences in which participants had position and color errors that exceeded three standard deviations were also excluded from the analysis (2.56% trials were removed on average for SS1, 6.54% trials for SS2, and 11.4% trials for SS4. Post these exclusions, the analysis comprised 29.2 (SD = 0.626), 27.1 (SD = 1.3), and 23.7 (SD = 2.64) sequences on average per participant for SSs 1, 2, and 4, respectively.

##### VWM utilization

Mean VWM utilization estimates were as follows: 0.848 (within-subject 95% CI [0.738, 0.956]), 1.137 [1.066, 1.209], and 1.06 [0.995, 1.130] for SSs 1, 2, and 4, respectively (F(2, 54) = 12.97, *p* < 0.001, η^2^ = 0.32). Post-hoc contrasts showed that the difference between SS1 and both SS2 and SS4 was significant (t(27) = 3.81, *p* < 0.001, η^2^ = 0.33), and that the difference between SS2 and SS4 was also significant (t(27) = − 2.22, *p* = 0.035, η^2^ = 0.15). An analysis of mean VWM utilization, which excludes steps in which participants have not placed any items in the reconstruction phase, was also conducted. The estimates of VWM utilization were 1 (within-subject 95% CI [0.89, 1.11]), 1.326 [1.26, 1.39], and 1.31 [1.23, 1.39] for SSs 1, 2, and 4, respectively (F(2, 58) = 19.02, *p* < 0.001, η^2^ = 0.41).

##### Task reliability

We calculated the Spearman-Brown corrected split-half reliabilities, based on odd vs. even trials, for all the dependent measures—utilization, color accuracy, and position accuracy similarly to the procedure in Experiment 1 (see Table 1).

##### Item positioning and color selection accuracy

Separate repeated-measures ANOVAs indicated that SS had no significant effect on the accuracy of color selection (F(2, 54) = 0.44, *p* = 0.694, η^2^ = 0.01) or item positioning (F(2, 54) = 0.55, *p* = 0.367, η^2^ = 0.04).

#### Individual differences

**Figure 4:**
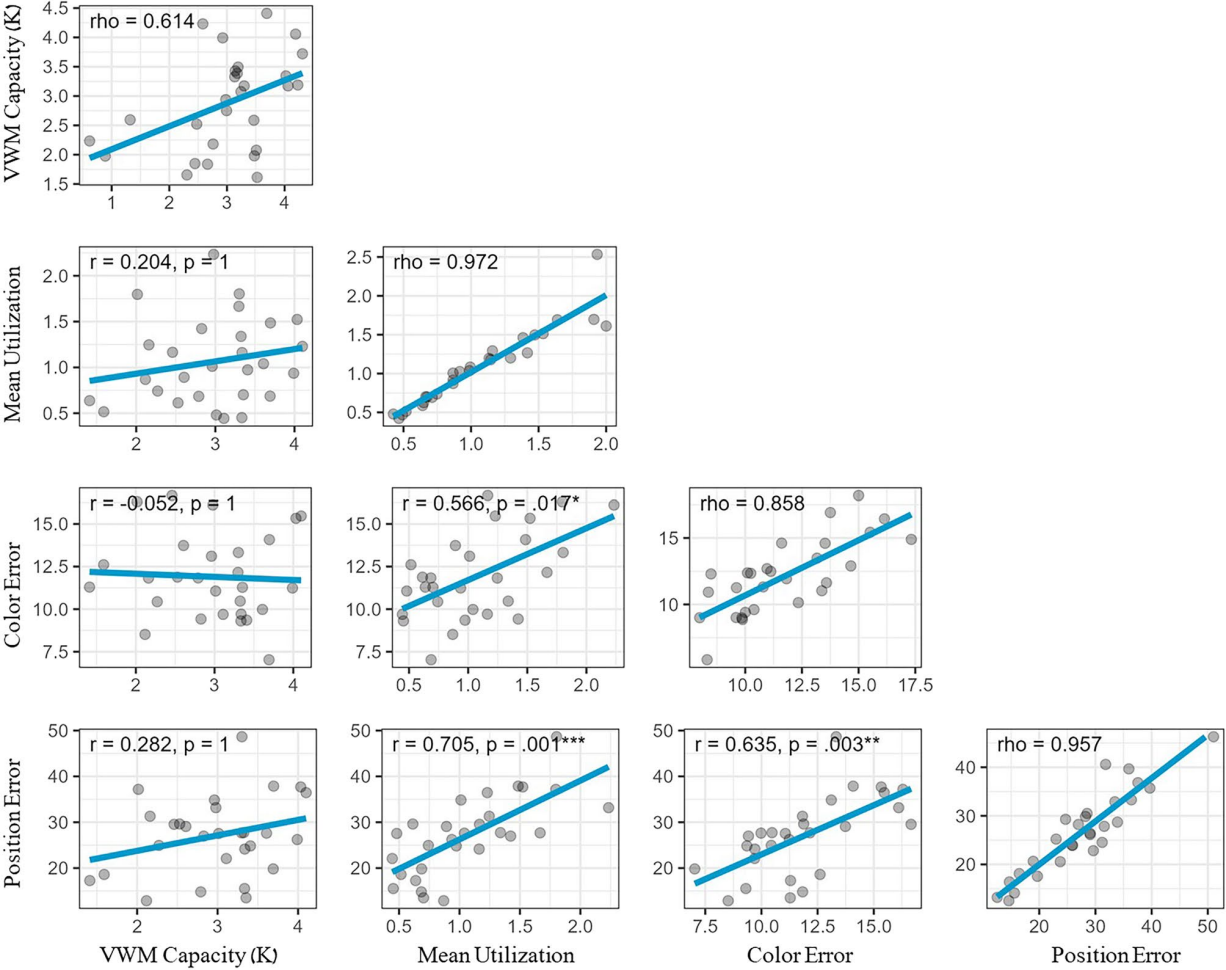
Correlation Matrix from Experiment 2 (SS4 trials only). The VWM capacity was assessed using the change detection task, while VWM utilization, item positioning error, and item color selection error were measured in the model reconstruction task. Each data point represents an individual participant. The main diagonal indicates the Spearman-Brown split-half reliability score (rho) for each metric based on odd vs. even trials.

The correlations depicting the relationships between the two tasks are illustrated in Fig. 4. p-values were Bonferroni-adjusted to the number of correlations As in Experiment 1, VWM capacity was uncorrelated with the utilization of VWM resources during the model reconstruction task (r = 0.204, p = 1). However, unlike the previous experiment, it was found that VWM capacity was uncorrelated with both the accuracy of item positioning (r = 0.282, p = 1) and the selection of corresponding colors (r = − 0.052, p = 1). In contrast, a significant correlation was identified between VWM utilization and both accuracy metrics (for color error r = 0.566, p = 0.017; for position error r = 0.705, p = 0.001). Specifically, participants who demonstrated higher utilization of their VWM resources tended to exhibit lower accuracy in their task performance.

### Discussion

The procedure for Experiment 2 closely resembled that of Experiment 1, only differing in its fixed model viewing time to better align with the change detection task. Despite this modification, participants still tended to underutilize their VWM capacity during the model reconstruction task. The average VWM utilization remained significantly lower than the VWM capacity estimation obtained from the change detection task. Moreover, individual differences between participants in the amount of VWM resources utilized were not explained by their VWM capacity limits, as we observed in the previous experiment.

In contrast to Experiment 1, which uncovered significant correlation between VWM capacity and color selection accuracy, this relationship was not replicated in the current experiment. Notably, the shortening of the viewing time seemed to nullify the correlation between VWM capacity and accuracy estimations. Intriguingly, correlations between VWM utilization and accuracy estimations emerged as statistically significant.

## General discussion

The present study aimed to develop a task that will allow us to investigate how individuals utilize their VWM resources in unforced behavior and to examine if VWM utilization is related to VWM capacity. To achieve this, we designed a novel task that allowed participants to control the amount of information they loaded into VWM in each step. The setting and stimuli in this task closely resembled those used in classic change detection and delayed estimation tasks, allowing the comparison of performance between the paradigms. Moreover, the simplicity of this task makes it suitable for online testing, in contrast to previous studies on the topic.

Our findings indicated that participants under-utilized their VWM capacity during the reconstruction of the target-model. Whereas the mean capacity was around 3 items, as typically observed in other studies, participants chose to utilize only around 1–1.5 items following each view of the model. This finding is consistent with previous results in the field, which have demonstrated a tendency for individuals to sample the environment repeatedly rather than fully exploiting their VWM capacity^9–12^. Notably, our model-reconstruction task eliminated the need for visual search during model replication, a process that could otherwise compete for VWM resources^9,10^, yet the inclination for under-utilization persisted. In addition, whereas the number of reconstructed items in each step was highly reliable in terms of individual differences, it was unrelated to VWM capacity.

Why do not people fully utilize their VWM resources when having the freedom to choose how many items to remember? The lack of correlation with VWM capacity hints that the degree of WM utilization does not reflect an ability to remember a certain amount of information but rather is, at least to some extent, a metacognitive decision that is under the participant’s control. This view is supported by Draschkow et al.’s^10^ study, which shows that WM utilization is not constant but reflects a tradeoff between reliance on WM and the physical effort required for reviewing the model. Three possible metacognitive strategies are viable.

### Item selection at encoding

This possibility holds that participants have control over the amount of information that enters their WM and can selectively broaden or narrow their encoded set at will. According to this strategy, when participants can freely choose the memoranda, they prefer encoding around one item even though, in principle, their WM can hold more items. This notion is compatible with discrete slots accounts of WM capacity^6,18^, which view the capacity limits as reflecting the number of maintained items. Accordingly, each item can either be maintained in WM or not, implying that under-utilization can take place by the decision to use only a subset of the available “slots”, or to allocate all the slots to a subset of the items in the target-model^19^. This predicts that unreported items are not maintained in WM, and that if participants were probed to report them, their performance would be at chance level.

A possible motivation for encoding only a subset of the items lies in the need for item-context associations/bindings when loading VWM with more than one item. When a single item is maintained, it is sufficient to remember the location and the color independently since their combination leads to a unique reconstruction of the item. However, when multiple items are retained, VWM must also maintain the bindings between each color to its location to prevent mis-binding (or “swap”) errors^20^. By choosing to focus on one item at a time, participants eliminate the need for such bindings. According to this view, VWM is not simply under-utilized when choice is permitted. Rather, it converges to one item due to the qualitatively different representation requirements in this case. A recent study from our lab supports this view by showing that working memory updating is costly in terms of response time when more than one item is maintained. However, updating working memory when only one item is stored is effortless and automatic^21^. Building on this work, we suggest that limiting encoding to a single item can be effective by avoiding the reliance on a costly and demanding updating process.

### Narrow resource allocation at encoding

An alternative view of strategic considerations during encoding emerges from theories that explain the capacity limitations of WM as reflecting a dynamic allocation of a shared resource^22^. According to this view, items are not either within WM or not. Rather, the precision of representation can flexibly vary among items. Under-utilization, in this case, may reflect a strategic narrowing of the resource allocation distribution among items, so that almost all the resources are devoted to one of the items, and the rest are distributed among other items. Unlike the previous account, the performance on a surprise recall test on one of the unreported items would not be at chance but would rather reflect maintenance with low precision (for a similar argument, see^23^).

### Partial report at retrieval

An alternative account to selective encoding is that our findings reflect processes that take place during retrieval. According to this possibility, participants have control over the number of reported items and prefer to place only the one(s) with the highest precision. This strategy could be a by-product of differential resource allocation at encoding, but could also reflect the loss of information during the recall phase. Specifically, even if the resources were evenly distributed among the items presented at encoding, precision differences might occur during the recall phase. The recall process is serial, requiring to place one item after the other. The passage of time and interference from the color wheel and the reconstruction array accumulates as participants report more items at a time, leading to the degradation of the memoranda. Accordingly, participants could strategically choose to report one item at a time, trading the number of reported items in exchange for better precision. Throughout the experiment, participants adopt a single-item report strategy, which might reflect an optimal tradeoff. While strategic considerations during recall are plausible, it remains to explain why performance converged to a reported set of only one item. For example, Adam et al.^24^ show that when probed to report all the items in the memory array, participants can recall around 3 items out of 6, with an above-chance precision. This finding suggests that our participants could, in principle, report more items if forced to do so. To better understand the role of selective reporting at retrieval, future studies should directly compare recall precision in our paradigm with that obtained in the whole-report procedure.

### Conclusion

To conclude, we believe that the study of free VWM resource utilization is an important and currently under-explored topic and that the paradigm developed in this study would make this research easy and accessible. Future studies should focus on the validity of VWM utilization, asking what the psychological constructs that correlate with this reliable measure are, as well as on group-level changes associated with development, aging, and neuropsychological conditions.

## Data Availability

Study materials, the data and their analysis code are available through OSF (https://osf.io/3cb2k/).
